# A Balanced Reciprocal Translocation T (X;20) in A Girl with Seizures and Intellectual Disability Disrupting ARHGEF9

**DOI:** 10.1186/1755-8166-7-S1-P59

**Published:** 2014-01-21

**Authors:** Usha R Dutta, Vijaya Kumar Pidugu, Vera M Kalscheuer, Ashwin B Dalal

**Affiliations:** 1 Centre for DNA Fingerprinting and Diagnostics, Hyderabad, India; 2 Max Planck Institute for Molecular Genetics, Berlin, Germany

**Keywords:** *ARHGEF9* gene, BCA, intellectual disability

## Background

Chromosomal aberrations are a significant cause of human disorders. The purpose of the present study is to characterize a balanced reciprocal translocation identified in a girl who presented with seizures, disturbed sleep, intellectual disability and focal hypopigmentation on the skin and identify the gene(s) involved.

## Materials and Methods

Several methods like GTG banding, array CGH, X-inactivation studies by methylation specific PCR for the human androgen- receptor gene (HUMARA), FISH (Fluorescence-in situ-hybridization) with whole chromosome paint probes (WCP) and with Bacterial Artificial Chromosome (BAC) clones from the regions of interest, RT-PCR expression analysis for *ARHGEF9* gene were used.

## Results

The chromosomal analyses revealed a translocation between the long arm of chromosome X and the short arm of chromosome 20 [46,X,t(X;20)(q12;p13)]. This result was confirmed by WCP FISH. Additionally, array CGH ruled out any gains or losses at the breakpoints or elsewhere in the genome. Also, X-inactivation studies by methylation specific PCR for HUMARA indicated skewed X-inactivation of the normal X chromosome. Breakpoint mapping of both derivative chromosomes was performed by serial FISH using BAC clones and RP11-943J20 from chromosome X showed split signals on patient derivative translocation chromosomes, indicating that this clone spanned the breakpoint. The breakpoint on 20p13 was mapped to a region of about 28 kb. Subsequent in silico analysis of the fine mapped breakpoint regions showed that on chromosome X, ARHGEF9 was likely disrupted by the chromosome rearrangement, whereas on chromosome 20 the breakpoint region does not seem to harbor a known gene. RT-PCR expression analysis of ARHGEF9 using RNA isolated from the patient’s lymphoblastoid cell line and a control suggested that in the patient the breakpoint maps between exons 1 and 2 of this gene. Further, the rearrangement has potentially resulted in fusion genes, suggested by the low expression of ARHGEF9 exons 2 to 10 in the patient.

## Conclusion

We have previously reported another chromosome rearrangement that truncated ARHGEF9 in a patient with epilepsy, anxiety, aggression, insomnia and learning and memory loss. Given the similar clinical phenotypes of both patients we propose that in the patient reported here ARHGEF9 loss of-function is likely to be the cause of disease.

